# Multiparametric MRI Characterization and Prediction in Autism Spectrum Disorder Using Graph Theory and Machine Learning

**DOI:** 10.1371/journal.pone.0090405

**Published:** 2014-06-12

**Authors:** Yongxia Zhou, Fang Yu, Timothy Duong

**Affiliations:** 1 Department of Radiology, University of Pennsylvania, Philadelphia, Pennsylvania, United States of America; 2 Research Imaging Institute, Departments of Ophthalmology, Radiology, Physiology, University of Texas Health Science Center, South Texas Veterans Health Care System, Department of Veterans Affairs, San Antonio, Texas, United States of America; University of Electronic Science and Technology of China, China

## Abstract

This study employed graph theory and machine learning analysis of multiparametric MRI data to improve characterization and prediction in autism spectrum disorders (ASD). Data from 127 children with ASD (13.5±6.0 years) and 153 age- and gender-matched typically developing children (14.5±5.7 years) were selected from the multi-center Functional Connectome Project. Regional gray matter volume and cortical thickness increased, whereas white matter volume decreased in ASD compared to controls. Small-world network analysis of quantitative MRI data demonstrated decreased global efficiency based on gray matter cortical thickness but not with functional connectivity MRI (fcMRI) or volumetry. An integrative model of 22 quantitative imaging features was used for classification and prediction of phenotypic features that included the autism diagnostic observation schedule, the revised autism diagnostic interview, and intelligence quotient scores. Among the 22 imaging features, four (caudate volume, caudate-cortical functional connectivity and inferior frontal gyrus functional connectivity) were found to be highly informative, markedly improving classification and prediction accuracy when compared with the single imaging features. This approach could potentially serve as a biomarker in prognosis, diagnosis, and monitoring disease progression.

## Introduction

Autism spectrum disorders (ASD) are a group of polygenetic developmental brain disorders with behavioral and cognitive impairment [1]. Affected individuals exhibit stereotypical repetitive movements, restricted interests, lack of impulse control, speech deficits, impaired intelligence and social skills compared to typically developing (TD) children [2]. The underlying neurological changes and their association with the clinical manifestations remain an active area of research.

Imaging studies have been instrumental in mapping aberrant connections in the brains of ASD children. Several studies revealed both structural and functional connectivity deficits in ASD [3], [4]. Structural connectivity derived from diffusion tensor imaging in ASD children demonstrate increased diffusivity and/or reduced fractional anisotropy in the long occipitofrontal fasciculus and inter-hemispheric corpus callosal (e.g., minor and major forceps) commissure [5], asymmetric and under-connected arcuate fasciculus language pathways [6], [7], as well as reduced cerebellar-cortical interconnectivity [8]. Functional connectivity MRI (fcMRI) shows abnormal dis-inhibition of some subcortical circuits [9], over- or under-connectivity in the superior temporal gyrus and amygdala [10]. The caudate nucleus, which plays an important role in behavior impulsivity control, novelty seeking trait, procedural skill learning, and memory function [11], has been reported to increase in volume and its functional connectivity to the cortices is enhanced in autistic adolescents and young adults [12], [13]. These abnormal caudate volume and caudate-cortical connectivity have been associated with repetitive autistic behaviors [12], [13].

A key clinical manifestation of ASD is impaired learning and social interactions [14]. Mirror mechanisms in frontoparietal and sensorimotor networks are involved in the imitation of others' actions in normal subjects [15], and have been suggested to be dysfunctional in children with ASD [16], [17]. In addition, neural networks underlying reflective mentalization [18] as well as emotional and interoceptive awareness [19] may be impaired in ASD. The default mode network (DMN) that consists of the medial frontal, posterior cingulate gyri and the temporo-parietal connection (which is involved in self awareness, social integration, learning and memory functions [20] and is usually deactivated under cognitive-demanded task conditions) has been found to be deficient in ASD children as well [21]. Furthermore such DMN dysfunction has been associated with decreased volumes of the structures involved [21].

The inferior frontal gyrus (IFG), which is involved in high-level memory, language production and comprehension, and learning interactions [22]–[24], has been reported to be abnormal in ASD children. Within the IFG, the pars opercularis plays a role in social interactions including imitation control [25], while the pars triangularis is associated with language comprehension [26]. Task-related fMRI results have demonstrated IFG abnormalities in ASD patients, including impaired social function and affective emotion [27], [28]. FcMRI shows decreased functional connectivity in IFG inhibitory circuits, consistent with disruption of mirror neurons and their projections involved in social communication and language development [29], [30].

Given the large amount of anatomical and functional connectivity imaging data, a unified model integrating multiple imaging measures may be helpful to better characterize ASD and predict the phenotypic outcome [31]. Small-world network analysis based on graph theory offers some advantages in analyzing complex and multidimensional data by providing global and local inter-regional modulation information of structural and functional brain networks [32]. A potential challenge is the possibility of retrieval redundancy (“curse of dimensionality”). Principal component analysis is a feature selection method that has been widely used to reduce dimensionality by identifying essential components in the data structure, while retaining most of the data variation (i.e., information) [33]. Maximal relevance and minimal redundancy (mRMR) [34] is another feature selection algorithm to reduce dimensionality which uses filters based on mutual information and correlation to account for maximal dependency and minimal redundancy.

The goals of this study were: i) to evaluate multi-parametric functional and structural MRI of the brain in ASD versus TD children using small-world network analysis based on graph theory to derive local and global efficiency, ii) to use machine-learning algorithms to evaluate the ability of these multiparametric MRI matrices to classify ASD versus TD groups, and iii) to employ machine-learning algorithms of these multiparametric MRI matrices to predict ASD clinical phenotypic outcomes, such as the revised autism diagnostic interview (ADI-R), autism diagnostic observation schedule (ADOS), and intelligence quotient (IQ) scores reflecting different aspects of social and learning abilities of subjects [35].

## Methods

### Participants and phenotypic information and MRI Imaging

Data was obtained from the multi-center Functional Connectome Project, which released MRI data of over 500 ASD patients (http://fcon_1000.projects.nitrc.org/indi/abide/). We downloaded the Autism Brain Imaging Data Exchange database from the COINS website (http://coins.mrn.org/) that hosted copies of data samples. In accordance with HIPAA guidelines and 1000 Functional Connectomes Project/INDI protocols, all datasets were de-identified. Consistent with the policies of the 1000 Functional Connectomes Project, data usage was approved for research purposes.

127 children with ASD (mean age: 13.5±6.0 years, 24.1% of female), 153 age- and gender-matched control TD children (mean age: 14.5±5.7 years, 24.8% of female) from the NYU/Yale/Stanford centers were selected for analyses based on age ranges. The full IQ scores were slightly lower in the ASD (104.3±18.9) compared to the TD group (111.7±14.4), with P = 0.001. Imaging data consisted of high spatial resolution 3D T1-based MPRAGE sequence (image size = 160×256×256, resolution = 1×1×1 mm^3^) and resting-state (RS)-fMRI obtained using a gradient echo EPI sequence (TR = 2000 msec, resolution  = 3×3×4 mm^3^, 180 volumes) with 33 slices covering the whole cerebrum.

The corresponding clinical data from five categories were also obtained, consisting of the ADOS scores, the ADI-R, and IQ tests (full scale and sub-functionality), social responsiveness scale (SRS) and Vinland adaptive behavior scale (VABS) with five domains. Detailed information regarding the phenotypic data was outlined in previously published articles [10], [14]. Briefly, the ADI-R is composed of 93 items focusing on the triadic functional domains, and administered via interview with categorical results provided. The full IQ tests evaluated both verbal IQ (VIQ) and performance IQ (PIQ). The ADOS is a semi-structured assessment of social affection and communication behaviors, using four modules to account for individual expressive language level and chronological age. The SRS rating scale measures the social ability of ASD children (including total and subdomains of cognition, communication, awareness and motivation). The VABS instrument consists of social and personal skills for everyday living, including subdomains such as interpersonal empathy and socialization.

### Image Processing and Data Analysis

*Preprocessing*: Standard preprocessing for RS-fMRI data analysis was performed using both FMRIB Software Library (FSL) and Analysis of Functional NeuroImages (AFNI) software packages with adapted scripts (http://www.nitrc.org/projects/fcon_1000). The steps included standard realignment, spatial Gaussian smoothing with full width at half maximum (FWHM) of 6 mm, band pass temporal filtering of 0.005–0.1 Hz, co-registration with the MPRAGE images, removal of nuisance signals (motion parameters, the global signal, and signals derived from cerebrospinal fluid and white matter), and non-linear transformation to Montreal Neurological Institute (MNI) standard space. For instance, in order to control for motion artifacts, regression and residual analyses of six motion parameters including three angular rotations (roll, pitch, and yaw in units of degrees) and three directional displacements (x,y,z in units of mm) were implemented in the preprocessing step.

### Volumetry analysis

Anatomical data were processed to derive the total supratentorial volume, regional subcortical volumes (45 regions), white matter (WM) volumes (70 regions), and cortical gray matter (GM) volumes (148 regions) using Freesurfer software (version 5.1.0) [36]. Regional volume comparisons were implemented after supratentorial volume normalization. The cortical thickness of 148 cortical regions was measured automatically based on minimal communication distance between the gray and white matter ribbon of the projected cortical surface [37]. Thickness measured with Freesurfer has been shown to be especially useful in characterizing the cortical folding and cytoarchitecture shaping in different age ranges [38].

### fcMRI analysis

The seed-based fcMRI was derived with seeds in the bilateral pars triangularis and opercularis regions of the IFG, the caudate, and the DMN core seed in the MNI 2 mm template space. These seed regions or networks were selected based on previously reported abnormalities in ASD [12], [13], [27], [28]. The total number of voxels (N) and average correlational z-value (Z) were computed from the functional connectivity map of each seed using a threshold of cluster corrected P<0.05. The statistical multiple-comparison correction was done based on Gaussian random field (GRF) theory and implemented with the FSL easythresh command using empirical minimum Z>2.3 threshold with cluster significance of P<0.05. In addition, to examine the functional interhemispheric coordination, voxel-mirrored homotopic correlations (VMHC) were derived. The global VMHC voxel number (minimum Z>2.3; cluster significance P<0.05, corrected) and average Z-value were then quantified [39].

### fALFF analysis

Fractional amplitude of low frequency fluctuation (fALFF) of the fcMRI data at 0.01–0.08 Hz, which reflects spontaneous neuronal or functional activity, was analyzed with adapted scripts in FSL and AFNI [39].

### Graph theory via small-world network analysis

Inter-regional correlations of individual fMRI time courses of 112 bilateral cortical and subcortical ROIs based on functional parcellation in FSL template space [40] were derived. Graph theory-based small-world network analysis for absolute and relative, local and global efficiency quantification at different connectivity sparsity levels, was then performed for each subject [32]. Group differences of mean local and global efficiency parameters derived from in-house software were compared with a 2-sample t-test. The inter-regional correlations of group-wise cortical thickness based on 148 bilateral cortical regions and volumetry measured with Freesurfer (148 cortical regions, as well as 115 subcortical and white matter regions) were also derived, and small world network analysis based on structural measures (including relative and absolute local as well as global efficiency parameters) was then performed and compared between the two groups.

### Integration model for two-group classification

To reduce possible classifier overfitting and improve generalization, feature selection was performed in two steps. First, principal component analysis was used to decompose the covariance matrix of the imaging features using the singular value decomposition program in Matlab (release 2010b; MathWorks, Natick, Mass) [33] after variance normalization. Then the number of sorted components based on singular values that contained 99% or 95% of the information from the covariance matrix of all features was determined. Finally, an advanced feature selection algorithm, based on mutual-information and integration of both mRMR criteria [34], was used to select imaging features based on the number of features (components) determined via principal component analysis.

A total of 22 quantitative local and global imaging features were generated from the structural and functional datasets based on preliminary data showing statistical differences between the two groups. These features include: i) volumes of 9 local subcortical regions (the bilateral caudate, thalami, pallidum, hippocampi and cerebellum), ii) 8 features from the fcMRI voxel number N and average Z values seeding from the bilateral caudate, bilateral IFG pars opercularis and triangularis, and DMN core seeds respectively, iii) 3 fALFF values (averaged values from the bilateral caudate, IFG pars opercularis, and IFG pars triangularis), and iv) global VMHC voxel number (corrected P<0.05) and average Z value (subtotal of 2 features).

To differentiate the two groups based on the selected imaging features, a total of 67 available classifiers, including support vector machine (SVM), Bayes network (BayesNet), radial basis function (RBF), and sequential minimal optimization (SMO) algorithms, were tested with batch-mode scripts developed in WEKA software (http://www.cs.waikato.ac.nz/ml/weka, version 3.6.7).

To evaluate each classifier, a cross validation method with different folds (numbering from 2 to 10) or percentage splits (from 10% to 90%) was applied to the training data set as well as to the whole dataset in WEKA software. After the dataset was randomly reordered and splitted into n folds of equal size, one fold was used for testing and the other n-1 folds were used for training the classifier for each iteration. The performances of these classifiers were further assessed in WEKA by using margin curves and threshold curves to show the cumulative probability of difference between true and false positives as a function of population size.

### Correlational analysis between imaging and phenotypic data

Quantitative analysis of single imaging features of regional volume, N and Z from fcMRI, as well as the combined selected imaging features were correlated with the clinical data (i.e., ADOS, ADI-R, SRS, VABS, and IQ tests including both full and sub-domain scores). Bonferroni adjustment was implemented by multiplying the original p-values with both the number of category tests (x5 in this study) and the number of sub-domain tests (e.g., x10 for ADOS, x3 for IQ, x5 for ADI-R, x7 for SRS and x15 for VABS) of each category.

### Integration model of imaging data for phenotypic prediction

To improve previous phenotypic correlation with multiparametric imaging data, regression analysis in WEKA (i.e., prediction of clinical data from imaging metrics) between the imaging features and clinical data was implemented. A total of 23 available advanced regression models were evaluated. These regressors were divided into seven families: Kstar, linear and non-linear ZeroR rule-based regressors, decision stump trees, and Gaussian process classifiers for full data sets. For example, Kstar was taken as an instance or memory based classifier using entropy and similarity functions with the advantage of updating models based on relevance to the previously established database [41].

## Results

### Volumetry analysis

**Figure 1:**
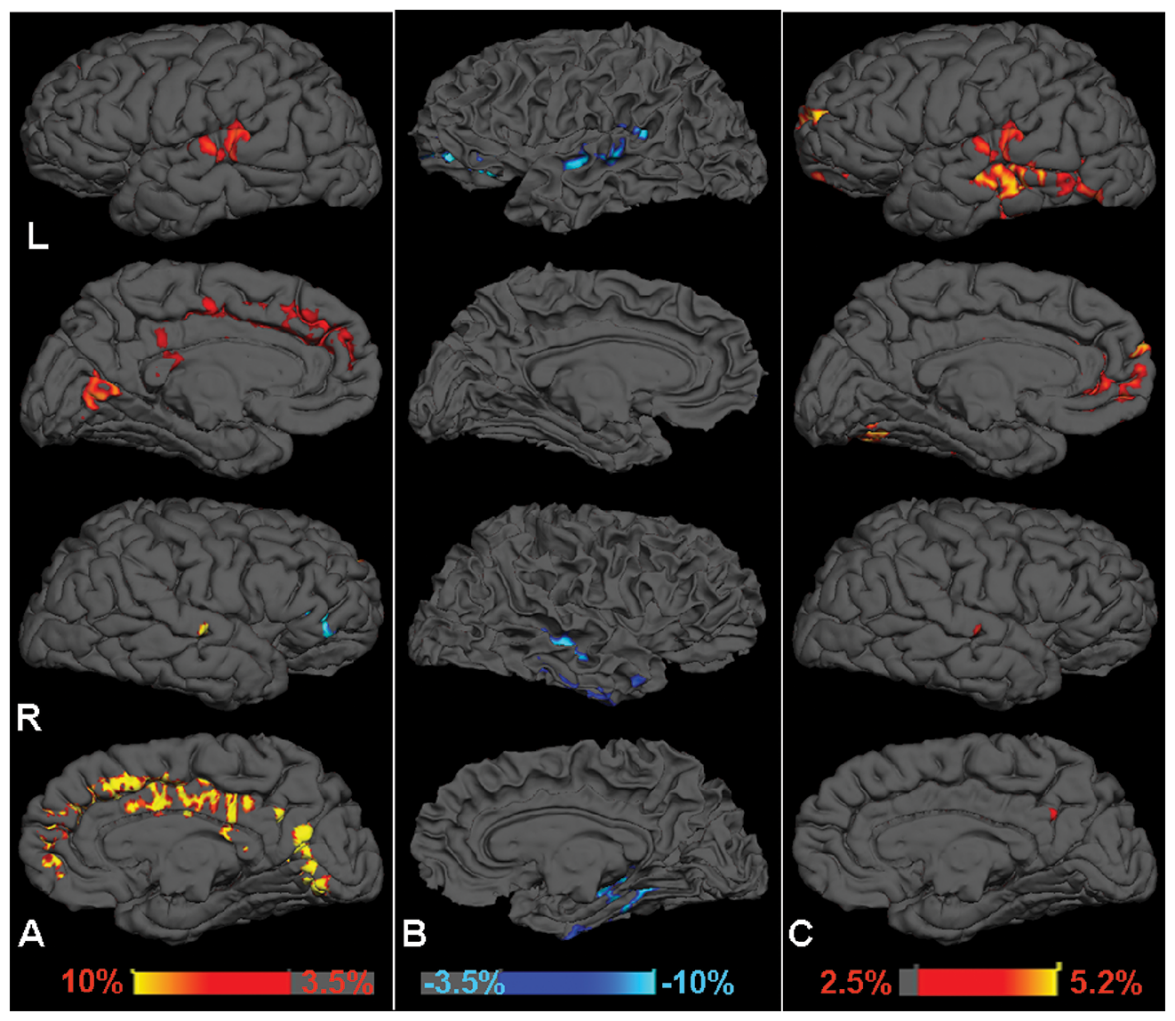
Regions with significant volume percentage increment (A, red color) in gray matter, and volume decrement in gray matter (A, blue color) as well as white matter volume reduction (B, blue color) in autism compared to controls (P<0.01). Significant cortical thickness increment in percentage change in autism compared to controls is shown in C with red color (P<0.01). Both medial and lateral views on sagittal surface in the left and right hemispheres of an individual control subject are shown for gray matter volume (A), white matter volume (B) and cortical thickness (C) comparison between autistic children and controls. The percentage change of volume in autism was calculated as the mean difference between volumes of two groups normalized with individual supratentorial volume and scaled by the mean normalized volume of control group.

Volume and cortical thickness differences between the ASD and TD groups are shown in **Figure 1** and **Table 1**. A key finding is that the ASD group demonstrated significantly larger GM volume (with exception of the right medial orbital olfactory region) but less WM volume compared to the TD group. Consistent with GM volumetric findings, the ASD group also showed significantly increased cortical thickness (**Table 2**).

**Table 1:** Significant regional volume differences (P<0.01) comparing children with autism spectrum disorder (ASD) to age-matched typically developing (TD) children after supratentorial volume normalization.

Region and Location	V1- TD Children	V2- ASD Children	P value (**)	Percentage change %(Δ1)
Frontal GM				
Left inferior	0.852	0.938	0.0068	10.094%
Left superior	18.97	20.05	0.0010*	5.693%
Right superior	18.18	19.12	0.0034*	5.171%
Right medial orbital olfactory	1.204	1.104	0.0034*	−8.306%
Frontal WM–left lateral orbitofrontal	5.761	5.562	0.0090*	−3.454%
Occipital GM				
Left middle	4.941	5.292	0.0056*	7.104%
Left superior	2.623	2.902	0.0002*	10.637%
Right superior	3.279	3.582	0.0017*	9.241%
Parietal GM				
Left postcentral	3.961	4.230	0.0064	6.791%
Right superior	5.482	5.888	0.0054*	7.406%
Right postcentral	3.996	4.319	0.0020*	8.083%
Right precuneus	6.495	7.031	0.0006*	8.253%
Parietal WM				
Left postcentral	5.771	5.483	0.0074	−4.990%
Right supramarginal	7.791	7.410	0.0054*	−4.890%
Temporal WM				
Left fusiform	5.811	5.567	0.0085	−4.199%
Right inferior	5.157	4.890	0.0023*	−5.177%
Right superior	5.779	5.543	0.0058*	−4.084%

Note-Data (V1, V2) are mean brain volumes after normalization to the supratentorial volume with a scale factor of 1000, no unit.

**Calculated with two-sample t test to obtain original p-value (shown with P<0.01) between two groups, and with Bonferroni multiple region correction (x8 factor given 4 brain lobes in two hemispheres).

*Indicates a significant difference with corrected P<0.05 after Bonferroni adjustment.

Δ1 Indicates percentage change between volume of ASD children (V2) and volume of TD children (V1), calculated as Δ1 =  (V2-V1)/V1*100%.

**Table 2:** Cortical thickness increment (in a degree of 2.5–5%) comparing autism spectrum disorder (ASD) children to age-matched typically developing (TD) children (P<0.01).

Region and Location	C1- TD Children	C2- ASD Children	P value (**)	Percentage change%(Δ2)
Frontal				
Left anterior cingulum	2.908	3.031	0.0008*	4.230%
Left mid-anterior cingulum	2.875	2.985	0.0004*	3.826%
Left transverse pole	2.982	3.119	0.0083	4.594%
Occipital				
Left middle	2.795	2.939	0.0002*	5.152%
Left superior	2.341	2.453	0.0056*	4.784%
Left lingual	2.215	2.308	0.0089	4.199%
Left middle lunatus	2.118	2.220	0.0026*	4.816%
Right lingual	2.287	2.383	0.0031*	4.198%
Right calcarine	2.129	2.231	0.0023*	4.791%
Parietal				
Left inferior angular	3.036	3.166	0.0016*	4.282%
Left inferior supramar	2.987	3.107	0.0004*	4.017%
Left rectus	2.807	2.903	0.0089	3.420%
Left post-lateral fissure	2.616	2.718	0.0018*	3.900%
Left occipital junction	2.428	2.531	0.0060*	4.242%
Left postcentral	2.338	2.444	0.0036*	4.534%
Right postcentral	2.311	2.423	0.0029*	4.846%
Temporal				
Left superior	2.691	2.792	0.0009*	3.753%
Right superior	2.731	2.806	0.0089	2.746%

Note-Data (C1, C2) are mean cortical thickness in mm.

**Calculated with two-sample t test to obtain original p-value (shown with P<0.01) between two groups, and with Bonferroni multiple region correction (x8 factor given 4 brain lobes in two hemispheres).

*Indicates a significant difference with corrected P<0.05 after Bonferroni adjustment.

Δ2 Indicates percentage change between cortical thickness of ASD children (C2) and cortical thickness of TD children (C1), calculated as Δ2  =  (C2–C1)/C1*100%.

### fcMRI analysis

**Figure 2:**
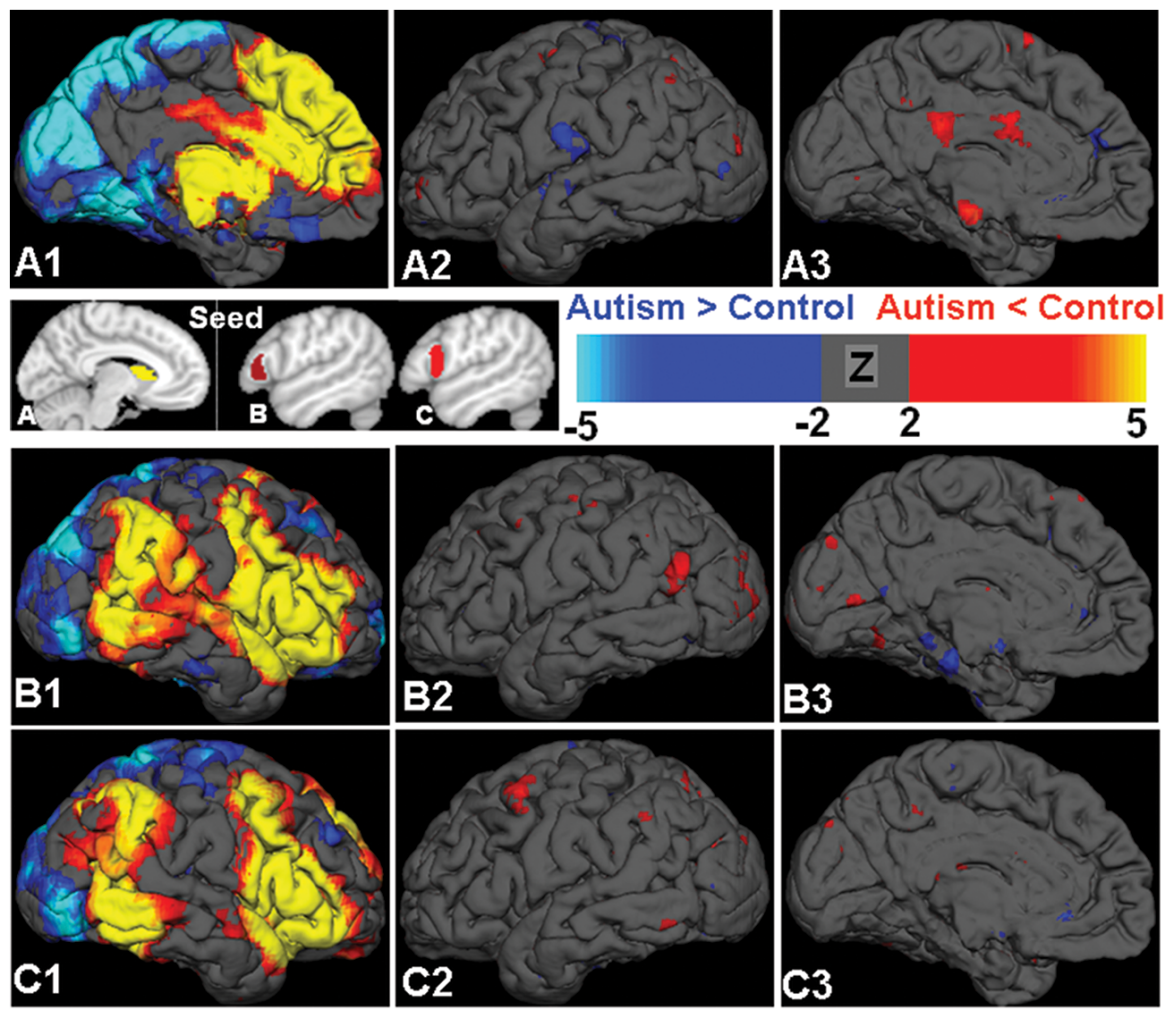
The functional connectivity network seeding from caudate and IFG region are demonstrated. In controls, the caudate-cortical network showed positive connections with the frontal and subcortical regions, and negative connections with the posterior visual and parietal areas (A1). The caudate-cortical network in children with ASD showed a primary reduction of cingulum and middle temporal connectivity (red color), but increased connectivity in several regions including the inferior and dorso-latero frontal areas (blue color, A2-A3). The functional connectivity network seeding from the IFG pars trianglaris in controls (B1) showed positive connections with the frontal and temporal regions and negative connections with the posterior visual and superior parietal areas. Difference of connectivity pattern comparing autism to controls seeded from pars trianglaris are shown in B2 and B3, with some reductions of visual and temporal connectivity in autism (red) and increments in the regions including medial temporal areas (blue). Functional connectivity seeding from IFG pars opercularis is shown in C1–C3, with primarily decreased connectivity in autism in the superior frontal regions. Seed regions are shown in the upper left images, and all statistical results were obtained with the threshold of minimal Z>2.3; cluster significance, P<0.05, corrected.

With seeding in the caudate, the ASD children demonstrated reduced functional connectivity in the cingulum and middle temporal cortex compared to TD children, but increased connectivity in several regions including the inferior and dorso-lateral frontal areas (**Figure 2A**). With seeding in the IFG pars triangularis, the ASD group showed reduced fcMRI of visual and temporal regions, but increased in the medial temporal areas (**Figure 2B**). With seeding from the IFG pars opercularis, the ASD group predominantly exhibited reduced functional connectivity with the superior frontal regions (corrected P<0.05; **Figure 2C**).

### Graph theory via small-world network analysis

**Figure 3:**
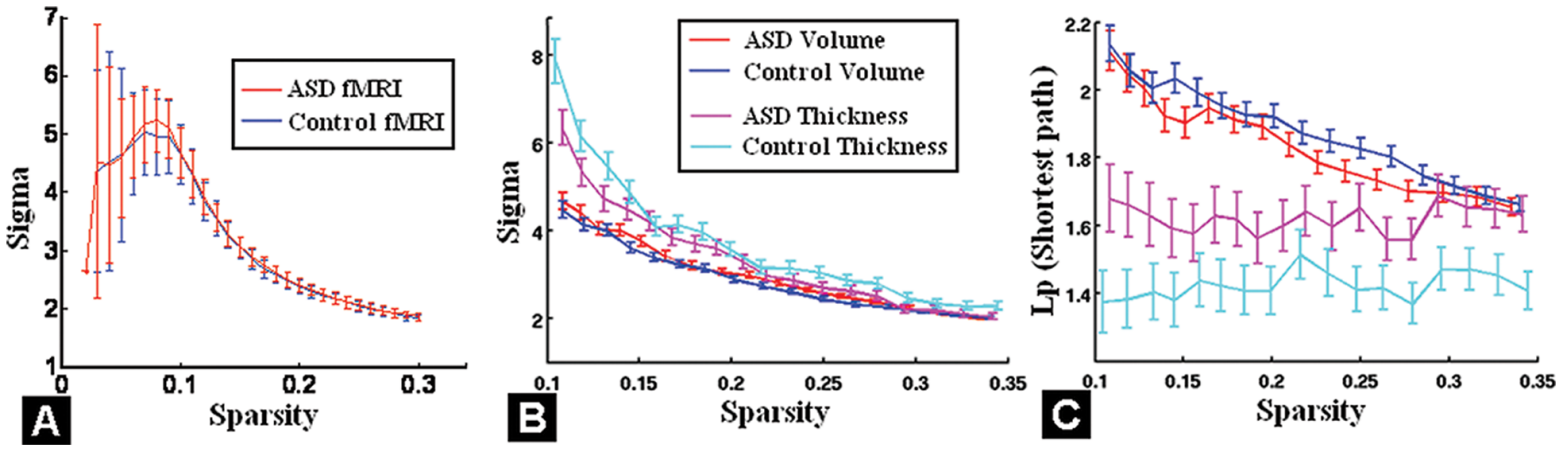
A: Small-world network analysis based on fMRI data showed similar efficiency (including both local and global) between children with ASD and TD controls. Small-worldness analysis based on regional volume (red and blue colors) showed no significant differences of total efficiency (B) and global efficiency (C) between children with ASD and controls neither. However, small-worldness analysis based on structural cortical thickness measure showed reduced total efficiency (B), specifically decreased global efficiency (i.e. shortest path length) (C) in ASD group (magenta color) compared to TD group (cyan color). Error bar denotes standard deviation at each sparsity level after scaling to a random network with the same number of degree.

Small-worldness analysis showed no statistically significant difference in the mean total (local and global) efficiency between ASD and TD children based on fcMRI (**Figure 3A**) and volumetric data (**Figure 3B,C**). However, there was reduced total efficiency in ASD children (**Figure 3B**) based on cortical thickness, especially in global efficiency, as indicated by increased shortest path lengths (**Figure 3C**).

### Classification of ASD and TD groups

**Figure 4:**
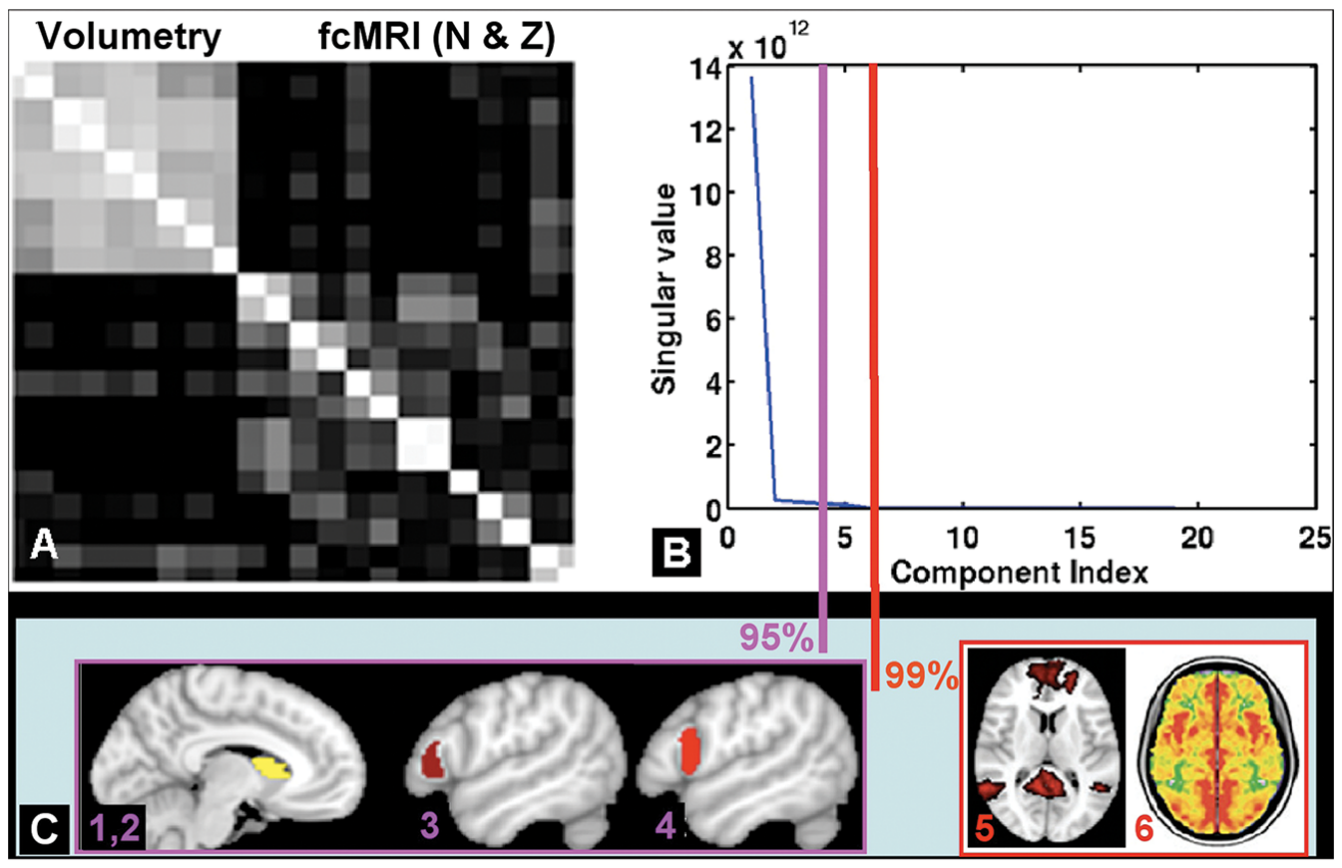
A: Co-variance matrix of 22 quantitative imaging features showed highly-correlated structural metrics (i.e. volumetry) of 9 regions, and strong association between average Z-value and voxel number N-value of fcMRI for each region (or global metric). B: principal component analysis decomposition of the covariance matrix showing most (>95%) data variation (information) was contained with 4 primary components (pink color) and 99% data information was contained in 6 primary components (red color). C: The four imaging features selected via mRMR criteria were right caudate volume (1), the fcMRI average Z values seeded from bilateral caudate (2), bilateral IFG pars opercularis (3) and IFG pars triangularis (4). Together with the four imaging features selected, the DMN average Z (5) and the VMHC average Z (6) were the other two imaging features selected for 99% criteria.

Co-variance matrix of the 22 imaging features (**Figure 4A**) showed correlated intra-module volumetry of 9 subcortical regions, as well as a strong association between the average Z-value and the voxel number of fcMRI, both regionally and globally. However, the inter-module correlation was not significant (r<0.3), suggesting volumetry and fcMRI provided complementary information. Singular value decomposition of the co-variance matrix (**Figure 4B**) found that 4 primary components accounted for >95% data variation, and 6 primary components accounted for >99% data variation. The four primary features were the right caudate volume, fcMRI Z values seeded from the bilateral caudate, IFG pars opercularis, and IFG pars triangularis; the additional two features were the mean Z score maps of the DMN and VMHC (**Figure 4C**).

**Figure 5:**
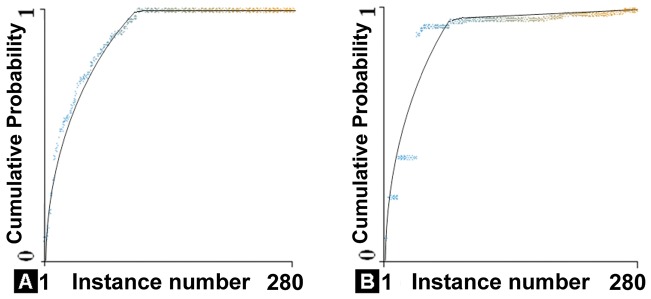
Margin curves showing the cumulative probability of difference between true positive (sensitivity) and false positive (1-specificity) as a function of instance number (i.e. subject number) for 4-feature classification (A) and 6-feature classification (B) based on full dataset. As expected, the 6-feature classifier performed slightly better than 4-feature classifier with faster convergence and more margins maintained.

For the 6 imaging features selected by the mRMR algorithm, the random tree classifier had the highest classification accuracy (100%) for correctly identifying ASD patients with the full dataset, and 70% accuracy for differentiating ASD patients from TD children using 80% percentage split cross validation. Based on the 4 imaging features, the random tree classifier also had the highest accuracy (98%) for the full dataset for two-group classification, with 68% accuracy for 10-fold cross validation. The performance of the 6-feature classifier was slightly better than that of the 4-feature classifier, with faster convergence and higher margins (i.e. better accuracy) maintained (**Figure 5**).

### Correlation Analysis

**Table 3** summarizes the correlation analysis of the quantitative imaging features with phenotypic scores in ASD children, with both uncorrected and multiple-comparison corrected p-values. Significant correlations were found: i) between the number of connections N based on fcMRI from the caudate and the full scale IQ in ASD children (r = −0.47, P = 0.034), ii) between caudate fcMRI N and ADOS scales (r≤−0.43, P<0.005), iii) between caudate fcMRI N and VABS social skill scores (r = 0.35, P<0.001), iv) between caudate fcMRI N and VABS interpersonal skills (r = −0.3, P<0.001), v) between the caudate connectivity strength, Z score and the full scale IQ (r = −0.47, P = 0.034), vi) between caudate fcMRI Z and VABS scores (P<0.001), vii) between the connections (both N and Z) from the two IFG seeds and ADOS tests (r≤−0.46, P≤0.04), and viii) between the functional connections from the two IFG seeds and VABS scores (P<0.001).

**Table 3:** Significant correlations between each single MRI quantitative metric and phenotypic tests in children with autism spectrum disorder (P<0.05), adjusted with number of available patients for each test.

MRI metric	Phenotypic Test	r	p
Caudate fcMRI (N)	ADOS social affection	−0.44	0.005
	ADOS research	−0.43	0.005
	VABS socialization	0.35	<0.0001*
	VABS interpersonal skill	−0.30	<0.0001*
	IQ (full)	−0.47	0.034
Caudate fcMRI (Z)	IQ (full)	−0.47	0.034
	VABS socialization	0.35	<0.0001*
	VABS interpersonal skill	−0.44	<0.0001*
	VABS daily living standard	0.26	0.0005*
IFG triangularis fcMRI (N)	ADOS module	−0.57	0.0016
	ADOS total	−0.46	0.014
	ADOS communication	−0.47	0.04
	ADOS research	−0.47	0.04
	VABS interpersonal skill	−0.43	<0.0001*
IFG opercularis fcMRI (N)	ADOS module	−0.64	0.0002
IFG opercularis fcMRI (Z)	ADOS module	−0.50	0.007
	VABS socialization	0.34	0.0001*
	VABS interpersonal skill	−0.25	0.0006*

Note: N is the total number of voxels and Z is the average correlational z-value computed from the functional connectivity map (fcMRI) of each seed using a threshold of cluster corrected P<0.05.

*Indicates a significant difference with corrected P<0.05 after Bonferroni adjustment, with multiplication factors determined by both the number of category tests applied (x5 in this study) and the number of sub-domain tests (e.g., x10 for ADOS, x3 for IQ, and x15 for VABS) of each category.

### Integration model for prediction

**Figure 6:**
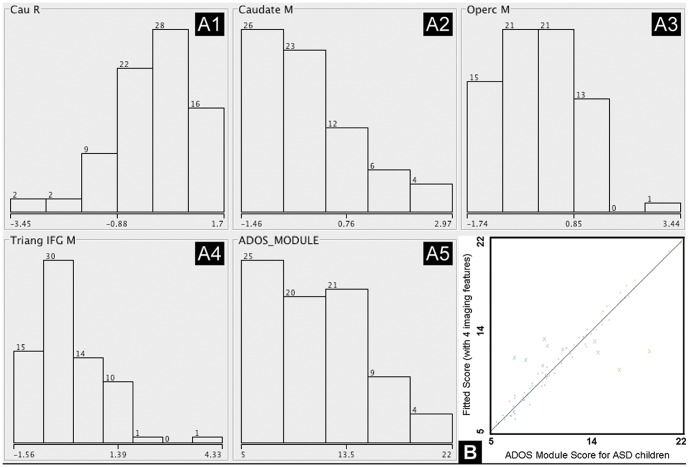
Kstar, an instance (similarity) based classifier for regression analysis had the greatest prediction power for ADOS module score for ASD children among all the regressors combining 4 selected imaging features. A1–A4: standardized histogram distribution showing x-axis as the z-scores of each feature and y-axis being the frequency. A1-right caudate volume, A2-average Z of caudate fcMRI, A3-average Z of IFG opercularis fcMRI, A4-average Z of IFG triangularis fcMRI. A5: phenotypic ADOS module score histogram distribution with x-axis as the original score and y-axis being the frequency. B: The correlation coefficient between integrated imaging features and ADOS module score improved largely from −0.64 based on single best imaging feature to 0.95 combining 4 imaging features. The fitted score using Kstar classifier with 4 selected imaging features showed a tight coupling (linear slope = 1; r = 0.95, P<0.0001) with the original ADOS module score with only a few outliers.

As for prediction, multiple combined imaging features in each domain showed improved correlations with clinical data compared to single imaging features for ASD children. For instance, fcMRI seeding from the pars opercularis of IFG showed a correlation coefficient of −0.64 between ADOS score and single imaging features (**Figure 6** **A1–A5**). The correlation coefficient was significantly improved with four selected imaging features to predict clinical phenotypic data. The fitted score using the Kstar classifier showed tight coupling (linear slope = 1, r = 0.95, P<0.0001) to the original ADOS module score, with only a few outliers (**Figure 6B**). Similarly, among all regressors, Kstar also showed the best predictive power for highly correlating with other phenotypic data including full IQ test (r = 0.94, P<0.0001) and ADI-R total score (r = 0.97, P<0.0001) when using the four as well as six essential imaging features.

## Discussion

We applied advanced automatic classification and machine learning algorithms to multiparametric functional connectivity and structural data (i.e., volumetry and cortical thickness) to improve characterization and prediction in autism spectrum disorders. The major findings of this study were: 1) small-world network analysis based on volumetry and fcMRI did not find differences between ASD and TD children, while small-world network analysis based on cortical thickness revealed reduced total and global efficiency in ASD, and 2) through advanced machine learning algorithms, four essential imaging features (caudate volume, caudate-cortical fcMRI and IFG pars opercularis as well as triangularis fcMRI) were able to accurately differentiate the two groups and were highly predictive of phenotypic features (e.g. IQ, ADOS and ADI scores) in ASD.

### Brain volume and thickness differences

Our findings of GM volume and cortical thickness increases and WM volume decreases in ASD compared to TD group are in general agreement with prior studies [42]–[44]. The increased cortical thickness in multiple regions throughout the cerebrum, together with decreased WM volumes in frontal and temporal regions, may represent a reduction in long-distance pathways with accompanying local GM volume increments. These changes could lead to a reduction in global efficiency, consistent with EEG/MEG results [45], [46] supporting the hypothesis of unbalanced networks in autism [47].

### Functional connectivity differences

Changes in caudate volume, caudate-cortical fcMRI, and IFG fcMRI were found to be highly predictive based on advanced machine learning algorithms. Most of the published literature on classification in autism is based on single imaging measures (such as volume, connectivity, or perfusion) [48]–[50]. Our integrated multiparametric model demonstrates markedly improved accuracy (i.e., sensitivity and specificity) for classification and prediction.

Consistent with task-based fMRI results [29], [30], we found altered IFG functional connectivity, suggesting deficits in the mirror mechanism in autistic children. Disrupted DMN fcMRI and altered interhemispheric functional connectivity are in agreement with previously reported studies in ASD [51], [52]. In addition, we found that caudate-cortical connectivity differed between ASD and TD children, and this metric correlated with full-scale IQ scores. A recent study reported aberrant functional connectivity from the caudate seed involving early developing brain areas and implicated developmental derangement of related functional brain circuits in ASD begin at a young age (from 7–14 years old) [14].

Small-world network analysis of fcMRI could not distinguish between ASD and TD groups, suggesting there might be functional compensatory mechanism in autism, including increased regional functional connectivity and reduced global, long-distance connectivity in ASD. These plasticity changes have been reported in other functional and structural connectome studies. For example, a recent fcMRI study found that there are changes in local efficiency in the posterior cingulum associated with autistic trait accompanied with changes in local tissue integrity in ASD as measured by diffusion tensor imaging [53]. The notion of re-routing of brain structural connectivity has also been reported in less severely cognitively impaired (IQ>70) ASD patients [54]. In particular, they found that these patients utilized more visuospatial processing networks as opposed to linguistically mediated pathways.

### Machine learning and prediction

Changes in caudate volume, caudate-cortical fcMRI, and IFG fcMRI were found to be highly predictive based on advanced machine learning algorithms. Most of the published literature on classification in autism has been based on single imaging measures (such as volume, connectivity, or perfusion) [48]–[50]. Our integrated multiparametric model demonstrates markedly improved accuracy (close to 100% sensitivity and specificity) for classification of ASD from TD groups.

The PCA and mRMR selected key features (i.e. right caudate volume, the average fcMRI Z values seeded from bilateral caudate, bilateral IFG opercularis and triangularis parts, the DMN average Z, and the VMHC average Z) are consistent with previous studies that found changes associated with ASD in each of the six imaging features. Our results based on machine learning algorithms also suggest that caudate volume and connectivity imaging features are important components in the composite score for ASD diagnosis.

Cross-validation minimizes classification error estimation bias and is thus primarily used for small sample datasets. As expected, the classification accuracy dropped from ∼100% for the full dataset to ∼70% with 10-fold or percentage-split cross-validation. The decreased accuracy in the cross-validation evaluation also reflected the heterogeneity of our data sample and a potential over-fitting problem. Nevertheless, the remarkable improvement in outcome prediction from multiple combined imaging features compared to any single imaging features (r≥0.94, P<0.0001) in all three phenotypic domains supports the notion that these key imaging features are reflective of the behavioral deficits in autism.

### Limitations and future works

Although we used substantially large numbers of classifiers and reported the optimal one, our results were limited by the selection criteria. Incorporating other factors such as different feature and subset selection criteria besides PCA and mRMR may improve the classifier performance in the future. The current quantitative fcMRI features include local and network-based features. Further expanding the fcMRI feature space, such as using whole-brain ROI-based fcMRI and fALFF of with dual regression template model [39], may reduce ROI-selection bias. Application of recently developed deep learning algorithms [55] to prevent feature overfitting problems could provide more comprehensive results. In order to minimize motion-related errors in RS-fMRI data, further investigation of motion parameters, such as frame displacement with dynamic temporal screening criteria, could be implemented in the future [56].

The regional parcellation with Freesurfer used in our study has been shown to be a robust method. However, the results of direct volumetry and cortical thickness could be affected by the normalization errors of projecting individual brains to a template brain, given the younger age range of our subjects compared to the averaged template. Implementing spatial normalization using age-specific templates in Freesurfer is likely important. An additional limitation is that the clinical phenotypic scales were obtained in the first years of life of participants, and not necessarily the period during which they underwent MRI scanning. This may potentially increase the possibility of mismatch between symptoms and brain features.

## Conclusion

This study applied advanced automatic classification and machine learning algorithms to multiparametric structural and functional connectivity data to improve characterization and prediction in autism spectrum disorders. A major finding of this study is that the small-world network analysis based on volumetry and fcMRI did not find differences between ASD and TD children, while the small-world network analysis analysis based on cortical thickness revealed reduced total and global efficiency in ASD. Moreover, we also found caudate volume, caudate-cortical fcMRI and IFG pars opercularis as well as triangularis fcMRI to be highly predictive of phenotypic features in ASD. Graph theory and machine learning analysis of multiparametric MRI may prove useful in identifying using imaging biomarkers for disease prognosis and monitoring disease progression in ASD.

## References

[1] Rapin I, Tuchman RF (2008) Autism: definition, neurobiology, screening, diagnosis. Pediatr Clin North Am 55: : 1129-1146, viii. 10.1016/j.pcl.2008.07.005 18929056

[2] Kang S, O'Reilly M, Rojeski L, Blenden K, Xu Z, et al (2013) Effects of tangible and social reinforcers on skill acquisition, stereotyped behavior, and task engagement in three children with autism spectrum disorders. Res Dev Disabil 34: 739–744. 23220050 10.1016/j.ridd.2012.10.007

[3] Uddin LQ, Supekar K, Menon V (2013) Reconceptualizing functional brain connectivity in autism from a developmental perspective. Frontiers in Human Neuroscience 7: 1–11. 23966925 10.3389/fnhum.2013.00458 PMC3735986

[4] Vissers ME, Cohen MX, Geurts HM (2012) Brain connectivity and high functioning autism: A promising path of research that needs refined models, methodological convergence, and stronger behavioral link. Neuroscience and Biobehavioral Reviews 36: 604–625. 21963441 10.1016/j.neubiorev.2011.09.003

[5] Travers BG, Adluru N, Ennis C, Tromp do PM, Destiche D, et al (2012) Diffusion tensor imaging in autism spectrum disorder: a review. Autism Res 5: 289–313. 22786754 10.1002/aur.1243 PMC3474893

[6] Lange N, Dubray MB, Lee JE, Froimowitz MP, Froehlich A, et al (2010) Atypical diffusion tensor hemispheric asymmetry in autism. Autism Res 3: 350–358. 21182212 10.1002/aur.162 PMC3215255

[7] Fletcher PT, Whitaker RT, Tao R, DuBray MB, Froehlich A, et al (2010) Microstructural connectivity of the arcuate fasciculus in adolescents with high-functioning autism. Neuroimage 51: 1117–1125. 20132894 10.1016/j.neuroimage.2010.01.083 PMC2966943

[8] Sivaswamy L, Kumar A, Rajan D, Behen M, Muzik O, et al (2010) A diffusion tensor imaging study of the cerebellar pathways in children with autism spectrum disorder. J Child Neurol 25: 1223–1231. 20179000 10.1177/0883073809358765

[9] Langen M, Schnack HG, Nederveen H, Bos D, Lahuis BE, et al (2009) Changes in the developmental trajectories of striatum in autism. Biol Psychiatry 66: 327–333. 19423078 10.1016/j.biopsych.2009.03.017

[10] Di Martino A, Kelly C, Grzadzinski R, Zuo XN, Mennes M, et al (2011) Aberrant striatal functional connectivity in children with autism. Biol Psychiatry 69: 847–856. 21195388 10.1016/j.biopsych.2010.10.029 PMC3091619

[11] Huang YY, Mei ZT (1983) [The role of the caudate nucleus in learning, memory and conditioning activity]. Sheng Li Ke Xue Jin Zhan 14: 356–358. 6678490

[12] Langen M, Durston S, Staal WG, Palmen SJ, van Engeland H (2007) Caudate nucleus is enlarged in high-functioning medication-naive subjects with autism. Biol Psychiatry 62: 262–266. 17224135 10.1016/j.biopsych.2006.09.040

[13] Turner KC, Frost L, Linsenbardt D, McIlroy JR, Muller RA (2006) Atypically diffuse functional connectivity between caudate nuclei and cerebral cortex in autism. Behav Brain Funct 2: 34. 17042953 10.1186/1744-9081-2-34 PMC1635430

[14] Di Martino A, Ross K, Uddin LQ, Sklar AB, Castellanos FX, et al (2009) Functional brain correlates of social and nonsocial processes in autism spectrum disorders: an activation likelihood estimation meta-analysis. Biol Psychiatry 65: 63–74. 18996505 10.1016/j.biopsych.2008.09.022 PMC2993772

[15] Hamilton AF (2013) Reflecting on the mirror neuron system in autism: a systematic review of current theories. Dev Cogn Neurosci 3: 91–105. 23245224 10.1016/j.dcn.2012.09.008 PMC6987721

[16] Gallese V, Rochat MJ, Berchio C (2012) The mirror mechanism and its potential role in autism spectrum disorder. DEVELOPMENTAL MEDICINE & CHILD NEUROLOGY 55: 15–22. 22924341 10.1111/j.1469-8749.2012.04398.x

[17] Rizzolatti G, Febbri-Destro M (2003) Mirror neurons: from discovery to autism. Exp Brain Res 2010: 223–237. 10.1007/s00221-009-2002-3 19760408

[18] Frith U (2001) Mind blindness and the brain in autism. Neuron 32: 969–979. 11754830 10.1016/s0896-6273(01)00552-9

[19] Ebisch SJ, Gallese V, Willems RM, Mantini D, Groen WB, et al (2011) Altered intrinsic functional connectivity of anterior and posterior insula regions in high-functioning participants with autism spectrum disorder. Human Brain Mapping 32: 1013–1028. 20645311 10.1002/hbm.21085 PMC6870194

[20] Fox MD, Raichle ME (2007) Spontaneous fluctuations in brain activity observed with functional magnetic resonance imaging. Nat Rev Neuroscience 8: 700–711. 17704812 10.1038/nrn2201

[21] Kennedy DP, Redcay E, Courchesne E (2006) Failing to deactivate: resting functional abnormalities in autism. Proc Natl Acad Sci U S A 103: 8275–8280. 16702548 10.1073/pnas.0600674103 PMC1472462

[22] Greenlee JD, Oya H, Kawasaki H, Volkov IO, Severson MA 3rd, et al (2007) Functional connections within the human inferior frontal gyrus. J Comp Neurol 503: 550–559. 17534935 10.1002/cne.21405

[23] Nixon P, Lazarova J, Hodinott-Hill I, Gough P, Passingham R (2004) The inferior frontal gyrus and phonological processing: an investigation using rTMS. J Cogn Neurosci 16: 289–300. 15068598 10.1162/089892904322984571

[24] Hsieh L, Gandour J, Wong D, Hutchins GD (2001) Functional heterogeneity of inferior frontal gyrus is shaped by linguistic experience. Brain Lang 76: 227–252. 11247643 10.1006/brln.2000.2382

[25] Rudie JD, Shehzad Z, Hernandez LM, Colich NL, Bookheimer SY, et al Reduced functional integration and segregation of distributed neural systems underlying social and emotional information processing in autism spectrum disorders. Cereb Cortex 22: 1025–1037. 10.1093/cercor/bhr171 PMC3328339 21784971

[26] Geva S, Jones PS, Crinion JT, Price CJ, Baron JC, et al The neural correlates of inner speech defined by voxel-based lesion-symptom mapping. Brain 134: 3071–3082. 21975590 10.1093/brain/awr232 PMC3187541

[27] Bastiaansen JA, Thioux M, Nanetti L, van der Gaag C, Ketelaars C, et al (2011) Age-Related Increase in Inferior Frontal Gyrus Activity and Social Functioning in Autism Spectrum Disorder. Biol Psychiatry 69: 8320838. 10.1016/j.biopsych.2010.11.007 21310395

[28] Depretto M, Davies MS, Pfeifer JH, Scott AA, Sigman M, et al (2006) Understanding emotions in others: mirror neuron dysfunction in children with autism spectrum disorders. Nat Neuroscience 9: 28–30. 16327784 10.1038/nn1611 PMC3713227

[29] Villalobos ME, Mizuno A, Dahl BC, Kemmotsu N, Muller RA (2005) Reduced functional connectivity between V1 and inferior frontal cortex associated with visuomotor performance in autism. Neuroimage 25: 916–925. 15808991 10.1016/j.neuroimage.2004.12.022 PMC3319340

[30] Lee PS, Yerys BE, Della Rosa A, Foss-Feig J, Barnes KA, et al (2009) Functional connectivity of the inferior frontal cortex changes with age in children with autism spectrum disorders: a fcMRI study of response inhibition. Cereb Cortex 19: 1787–1794. 19068486 10.1093/cercor/bhn209 PMC2722789

[31] Di Martino A, Yan C-G, Li Q, Denio E, Castellanos FX, et al (2013) The autism brain imaging data exchange: towards a large-scale evaluation of the intrinsic brain architecture in autism. Molecular Psychiatry 2013: 1–9. 10.1038/mp.2013.78 PMC4162310 23774715

[32] Zhou Y, Lui YW (2013) Small world properties in mild cognitive impairment and early Alzheimer's disease. ISRN Geriatrics 2013: 542080. 25414852 10.1155/2013/542080 PMC4235771

[33] Elliott MA, Walter GA, Swift A, Vandenborne K, Schotland JC, et al (1999) Spectral quantitation by principal component analysis using complex singular value decomposition. Magn Reson Med 41: 450–455. 10204865 10.1002/(sici)1522-2594(199903)41:3<450::aid-mrm4>3.0.co;2-9

[34] Peng H, Long F, Ding C (2005) Feature selection based on mutual information: criteria of max-dependency, max-relevance, and min-redundancy. IEEE Trans Pattern Anal Mach Intell 27: 1226–1238. 16119262 10.1109/TPAMI.2005.159

[35] Buitelaar JK, van der Wees M, Swaab-Barneveld H, van der Gagg RJ (1999) Verbal memory and performance IQ predict theory of mind and emotion recognition ability in children with autistic spectrum disorders and in psychiatric control children. J Child Psychol Psychiatry 40: 869–881. 10509882

[36] Fischl B (2012) FreeSurfer. Neuroimage 62: 774–781. 22248573 10.1016/j.neuroimage.2012.01.021 PMC3685476

[37] Fischl B, Dale AM (2000) Measuring the thickness of the human cerebral cortex from magnetic resonance images. Proc Natl Acad Sci U S A 97: 11050–11055. 10984517 10.1073/pnas.200033797 PMC27146

[38] Sowell ER, Peterson BS, Kan E, Woods RP, Yoshii J, et al (2007) Sex differences in cortical thickness mapped in 176 healthy individuals between 7 and 87 years of age. Cereb Cortex 17: 1550–1560. 16945978 10.1093/cercor/bhl066 PMC2329809

[39] Zhou Y, Milham M, Zuo XN, Kelly C, Jaggi H, et al (2013) Functional Homotopic Changes in Multiple Sclerosis with Resting-State Functional MR Imaging. AJNR Am J Neuroradiol 34: 1180–1187. 23348760 10.3174/ajnr.A3386 PMC3707620

[40] Stark DE, Margulies DS, Shehzad ZE, Reiss P, Kelly AM, et al (2008) Regional variation in interhemispheric coordination of intrinsic hemodynamic fluctuations. J Neurosci 28: 13754–13764. 19091966 10.1523/JNEUROSCI.4544-08.2008 PMC4113425

[41] Gagliardi F (2011) Instance-based classifiers applied to medical databases: diagnosis and knowledge extraction. Artif Intell Med 52: 123–139. 21621400 10.1016/j.artmed.2011.04.002

[42] Ecker C, Ginestet C, Feng Y, Johnston P, Lombardo MV, et al (2013) Brain surface anatomy in adults with autism: the relationship between surface area, cortical thickness, and autistic symptoms. JAMA Psychiatry 70: 59–70. 23404046 10.1001/jamapsychiatry.2013.265

[43] Ecker C, Stahl D, Daly E, Johnston P, Thomson A, et al (2009) Is there a common underlying mechanism for age-related decline in cortical thickness? Neuroreport 20: 1155–1160. 19690502 10.1097/WNR.0b013e32832ec181

[44] Hardan AY, Muddasani S, Vemulapalli M, Keshavan MS, Minshew NJ (2006) An MRI study of increased cortical thickness in autism. Am J Psychiatry 163: 1290–1292. 16816240 10.1176/appi.ajp.163.7.1290 PMC1509104

[45] Khan S, Gramfort A, Shetty NR, Kitzbichler MG, Ganesan S, et al (2013) Local and long-range functional connectivity is reduced in concert in autism spectrum disorders. Proc Natl Acad Sci U S A 110: 3107–3112. 23319621 10.1073/pnas.1214533110 PMC3581984

[46] Peters JM, Taquet M, Vega C, Jeste SS, Sanchez Fernandez I, et al (2013) Brain functional networks in syndromic and non-syndromic autism: a graph theoretical study of EEG connectivity. BMC Med 11: 54. 23445896 10.1186/1741-7015-11-54 PMC3626634

[47] Walsh CA, Morrow EM, Rubenstein JL (2008) Autism and brain development. Cell 135: 396–400. 18984148 10.1016/j.cell.2008.10.015 PMC2701104

[48] van der Zande FH, Hofman PA, Backes WH (2005) Mapping hypercapnia-induced cerebrovascular reactivity using BOLD MRI. Neuroradiology 47: 114–120. 15616848 10.1007/s00234-004-1274-3

[49] Wang H, Chen C, Fushing H (2012) Extracting multiscale pattern information of fMRI based functional brain connectivity with application on classification of autism spectrum disorders. PLoS One 7: e45502. 23056205 10.1371/journal.pone.0045502 PMC3466274

[50] Duchesnay E, Cachia A, Boddaert N, Chabane N, Mangin JF, et al (2011) Feature selection and classification of imbalanced datasets Application to PET images of children with autistic spectrum disorders. NeuroImage 57: 1003–1014. 21600290 10.1016/j.neuroimage.2011.05.011

[51] Anderson JS, Druzgal TJ, Froehlich A, DuBray MB, Lange N, et al (2011) Decreased interhemispheric functional connectivity in autism. Cereb Cortex 21: 1134–1146. 20943668 10.1093/cercor/bhq190 PMC3077433

[52] Assaf M, Jagannathan K, Calhoun VD, Miller L, Stevens MC, et al (2010) Abnormal functional connectivity of default mode sub-networks in autism spectrum disorder patients. Neuroimage 53: 247–256. 20621638 10.1016/j.neuroimage.2010.05.067 PMC3058935

[53] Jakab A, Emri M, Spisak T, Szema-Nagy A, Beres M, et al (2013) Autistic traits in neurotypical adults: correlates of graph theoretical functional network topology and white matter anisotropy patterns. PLoS One 8: 1–21. 10.1371/journal.pone.0060982 PMC3618514 23593367

[54] Sahyoun CP, Belliveau JW, Soulieres I, Schwartz S, Mody M (2010) Neuroimaging of the functional and structural networks underlying visuospatial vs. linguistic reasoning in high-functioning autism. Neuropsychologia 48: 86–95. 19698726 10.1016/j.neuropsychologia.2009.08.013 PMC2795068

[55] Hinton GE, Osindero S, Teh YW (2006) A fast learning algorithm for deep belief nets. Neural Comput 18: 1527–1554. 16764513 10.1162/neco.2006.18.7.1527

[56] Zhou Y, Lui YW, Zuo X-N, Milham MP, Reaume J, et al (2014) Characterization of thalamo-cortical association using amplitude and connectivity of functional MRI in mild traumatic brain injury. J Magn Reson Imaging 39: 1558–1568. 24014176 10.1002/jmri.24310 PMC3872273

